# Unravelling the Epigenetic Code: DNA Methylation in Plants and Its Role in Stress Response

**DOI:** 10.3390/epigenomes8030030

**Published:** 2024-08-08

**Authors:** Emanuela Talarico, Alice Zambelli, Fabrizio Araniti, Eleonora Greco, Adriana Chiappetta, Leonardo Bruno

**Affiliations:** 1 Department of Biology, Ecology and Earth Sciences (DiBEST), University of Calabria, 87036 Rende, Italy; emanuela.talarico@unical.it (E.T.); eleonora.greco@unical.it (E.G.); adriana.chiappetta@unical.it (A.C.); 2 Department of Agricultural and Environmental Sciences—Production, Landscape, Agroenergy, University of Milan, 20133 Milan, Italy; alice.zambelli@unimi.it (A.Z.); fabrizio.araniti@unimi.it (F.A.)

**Keywords:** abiotic stress, DNA methylation, epigenetic, grow plasticity

## Abstract

Environmental stress significantly affects plant growth, development, and survival. Plants respond to stressors such as temperature fluctuations, water scarcity, nutrient deficiencies, and pathogen attacks through intricate molecular and physiological adaptations. Epigenetic mechanisms are crucial in regulating gene expression in response to environmental stress. This review explores the current understanding of epigenetic modifications, including DNA methylation, and their roles in modulating gene expression patterns under environmental stress conditions. The dynamic nature of epigenetic modifications, their crosstalk with stress-responsive pathways, and their potential implications for plant adaptation and crop improvement are highlighted in the face of changing environmental conditions.

## 1. Introduction

### 1.1. DNA Methylation Mechanism in Plants

DNA methylation is a conserved epigenetic mark that regulates various processes, including gene expression, genome stability, and gene imprinting. Consequently, disruptions in DNA methylation can lead to developmental abnormalities [1].

Methylations on the fifth position of the pyrimidine ring of cytosine (*5*-methylcytosine [5mC]) and the sixth position of the purine ring of adenine (*N*6-methyldeoxyadenosine [6mA]) are the most abundant DNA modifications in prokaryotes and eukaryotes [2], as also revised in [3].

In mammals, DNA methylation predominantly occurs at CG sites, with non-CG methylation restricted to specific tissues, such as embryonic stem cells (ESCs) [4]. Conversely, in higher plants, both CG and non-CG methylation (including DNA methylation in CHG (symmetric) and CHH (asymmetric) contexts, where H = A, C, or T) are commonly found [1]. It is highly enriched over heterochromatic transposable elements (TEs) where it plays a crucial role in silencing their expression at the transcriptional level, a process known as transcriptional gene silencing (TGS). DNA methylation is not confined to gene promoter regions but also occurs within coding regions.

DNA methylation can also trigger TGS when present in gene regulatory regions. Additionally, methylation of intronic TEs and repeats has been shown to affect mRNA processing mechanisms such as alternative splicing (AS) and alternative polyadenylation (APA) [1]. In some cases, DNA methylation can also promote gene expression, a process partially mediated by the DNA methyl-readers SU(VAR)3-9 homologs (SUPPRESSOR OF VARIEGATION 3–9 HOMOLOG) SUVH1 and SUVH3 [5,6]. Consequently, numerous examples of natural and induced epialleles alter their expression in response to methylation changes, impacting various physiological processes [1]. DNA methylation, primarily in the CG context, is also found in gene bodies in many plant species. While its function in this context is poorly understood [7], a recent study reported a role in suppressing intragenic antisense transcripts [8]. In plants, there are three primary processes of DNA methylation: maintenance methylation (methylation of hemimethylated symmetrical sequences), de novo methylation (methylation of previously unmethylated cytosines), and DNA demethylation (reversal of the methylation state). The plant methylome is primarily sustained during DNA replication and cell division by DNA methyltransferases (Dnmts), including maintenance and de novo methylases [9]. The DNA methylation pattern can be faithfully transmitted across generations, ensuring transgenerational epigenetic inheritance [10,11,12]. Various enzymatic families and subfamilies of Dnmts are responsible for establishing and maintaining DNA methylation patterns in plants and animals. These enzymes act on different sequence contexts and are involved in maintenance and de novo methylation processes. In plants specifically, the preservation of DNA methylation at CpG dinucleotides is maintained by METHYLTRANSFERASE1 (MET1), an ortholog of the mammalian DNA METHYLTRANSFERASE 1 (DNMT1) [13,14,15,16]. Meanwhile, the maintenance of CHG methylation is regulated by plant-specific enzymes, CHROMOMETHYLASE 2 and 3 (CMT2 and CMT3) [17]. As for asymmetric CHH methylation, it is established through de novo methylation activities conducted by CMT3 and DOMAINS REARRANGED METHYLTRANSFERASEs (DRMs), which share orthology with mammalian Dnmt3a/b methyltransferases [17,18,19,20,21]. Furthermore, DNA demethylating enzymes (DMLs) can actively remove DNA methylation, contributing to the methylome dynamic nature [22,23,24].

The interplay between two key epigenetic marks, DNA methylation and H3K27me3 (Histone H3 Lysine 27 trimethylation) histone modification, and their role in shaping plant development has been demonstrated. Several molecular mechanisms influenced by DNA methylation were investigated by using various DNA-methylation-related mutants in the model species *Arabidopsis thaliana* [25].

Specifically, Forgione et al. [25] used the triple mutant *domains rearranged methyltransferases1, domains rearranged 2 chromomethylase 3 drm1 drm2 cmt3.11* (*ddc*) of *Arabidopsis*, which is impaired in both maintaining and establishing DNA methylation patterns. These mutations led to phenotypic alterations, including abnormal embryo development, agravitropic root growth, reduced rosette area, and individual curly-shaped leaves. Furthermore, the authors identified an altered leaf differentiation pattern characterized by smaller epidermal cells and an increased stomatal density compared to wild-type plants [25,26]. These developmental abnormalities were closely linked to disruptions in auxin pathways, including hormone synthesis, transport, and signaling [25]. In addition, organ-specific regulation of auxin-related genes, with some showing a direct influence of gene-specific methylation patterns, was observed [25,26]

These changes are attributed to the upregulation of the SUPPRESSOR OF DRM1 DRM2 CMT3 (SDC) F-box gene due to the loss of DNA methylation in its promoter region [27].

### 1.2. DNA Methylation and Plant Response to Environmental Challenges

Plants are sessile organisms exposed to many environmental challenges that affect their growth, development, and survival. These challenges are often due to abiotic stresses like drought, extreme temperatures, high salinity, and nutrient limitations, which seriously threaten their survival [28]. To overcome the effects of these stresses, plants have developed a remarkable variety of adaptive strategies. Among them, epigenetic modifications have become important components of plant environmental stress responses.

The term ‘epigenetics’ was coined by Waddington in the mid-20th century [29] by merging the terms ‘epigenesis’ and ‘genetics’ to describe the study of plant phenotypic traits. This area of research aimed to understand how genes and encoded proteins interact to determine the plasticity of these traits. However, recent advances in molecular biology have led to a more focused definition. This exploration aimed to understand how genes and their products interact causally to shape these traits [30]. Indeed, epigenetic mechanisms involve heritable changes in gene expression without alterations to the underlying DNA sequence [31]. These modifications can influence gene regulation and phenotypic traits by modifying the structure and accessibility of chromatin, thereby affecting transcriptional activity. Key epigenetic modifications in plants include DNA methylation, histone modifications, and RNA interference (RNAi) [32,33,34]. Similarly to RNAi, microRNA (miRNA) can also regulate the expression of genes before and after transcription, playing a crucial role in plant development, particularly in reproductive processes [35,36,37,38,39,40]. For example, in *Olea europaea* L., several novel miRNA have been characterized to be involved in fruit ripening mediated by the flavonoid and anthocyanin biosynthetic pathways [41]. DNA methylation is among the earliest discovered and extensively studied regulatory mechanisms in epigenetics. It is widely recognized for its relative stability and heritability, playing a crucial role in various biological processes, including gene expression, transposable element activity, and genomic imprinting [42].

DNA methylation is a powerful spatial and temporal regulator of gene expression that responds to changing environmental conditions. In particular, DNA methylation, along with other epigenetic modifications, facilitates chromatin remodeling processes and regulates gene expression. Consequently, DNA methylation substantially shapes plant diversity and influences developmental processes [42,43,44,45,46].

Various techniques are available for the detection of DNA methylation. These encompass methods that rely on antibodies or specific proteins with an affinity for methylcytosine, including methylated DNA immunoprecipitation (MeDIP) and the use of methyl Cpg-binding domain (MBD) proteins (MBDCap) [47]. Alternatively, approaches involving methyl-sensitive restriction enzymes, such as methyl-sensitive amplification polymorphism (MSAP) [48,49] and methods centered on the treatment of DNA with sodium bisulfite, like whole genome bisulfite sequencing (WGBS), are also employed [50].

The choice of method depends on the required resolution and cost considerations. Techniques based on affinity enrichment are ideal for rapid, large-scale investigations with lower resolution. In contrast, restriction enzyme methods are better suited for targeted, site-specific studies. Meanwhile, bisulfite conversion techniques excel in high-resolution analysis.

External cues, particularly environmental stressors, significantly influence DNA methylation patterns and are crucial in mediating plant responses to these challenges [51,52,53,54]. To survive and thrive in adverse conditions, plants use DNA methylation, a chemical modification of their genetic material, as a dynamic tool to fine-tune gene expression and adapt to changing environmental conditions [51,52,53,54].

The relationship between DNA methylation and plant response to environmental stress is intricate and versatile. It allows plants to customize their genetic responses to specific stressors, optimizing their survival and reproduction chances. This review explores the multifaceted interactions between DNA methylation and environmental stress responses in plants, highlighting how epigenetic marks influence the molecular mechanisms governing adaptation to a variable and often hostile environment.

Specific examples illustrate the importance of DNA methylation in plant stress responses. For instance, prolonged drought conditions can trigger DNA methylation in some plant species to silence genes responsible for water-consuming processes, thereby conserving water and ensuring survival, as also revised in [55,56,57]. Conversely, during extreme cold, DNA demethylation can activate genes that produce antifreeze proteins, preventing ice crystal formation within plant cells [58].

Furthermore, DNA methylation can facilitate transgenerational stress memory. The plant progeny can inherit stress-induced methylation changes, providing an early advantage when facing similar environmental challenges [59,60]. These examples demonstrate the fascinating interplay between DNA methylation and plant responses to environmental stress. They show how epigenetic modifications enable plants to fine-tune their genetic responses and enhance their chances of survival. This knowledge raises hopes for developing stress-tolerant crops and promoting sustainable agriculture in an era of environmental uncertainty.

## 2. DNA Methylation in Regulating Plant Response to Cold Stress

Plant growth and development and crop yield are extremely sensitive to temperature variations and can be affected by freezing or very low temperatures. To counter cold stress, plants employ various mechanisms to mitigate the potential damage caused by low temperatures [61]. Changes in DNA methylation status are a key way in which gene expression is regulated in response to cold stress [62,63].

Numerous studies unravel the importance of DNA methylation, particularly 5mC and 6mA, in regulating transcriptional responses to cold stress [62,63,64]. 6mA DNA methylation has recently emerged as a potential novel epigenetic marker in eukaryotes, including the dicot model *Arabidopsis thaliana* [65]. It has been demonstrated that high-resolution 6mA methylomes of two different rice cultivars, Nipponbare (Nip; Japonica) and 93-11 (Indica), showed a positive correlation between 6mA levels and the expression of stress-responsive genes, potentially influencing the stress tolerance [64].

5mC stands out as the most extensively studied epigenetic modification in cold stress. It plays a crucial role in the ability of plants to transmit cold-stress-induced DNA methylation changes both within a single generation and across generations, allowing them to respond more effectively to cold stress in subsequent encounters [66].

Cold exposure significantly reduced DNA methylation levels in sugar beet (*Beta vulgaris*) [67]. In apple (*Malus × domestica* Borkh.) [68], total methylation decreased under high-chill conditions but not significantly under low-chill conditions. Cold-tolerant genotypes prevented the accumulation of hydrogen peroxide (H_2_O_2_), as indicated by the reduction in malondialdehyde and electrolyte-leakage levels, known as indices of damage, compared to sensitive genotypes [69]. During prolonged cold stress, tolerant genotypes show greater demethylation bands changes than sensitive genotypes. This suggests a higher activation of genes putatively involved in cold stress response in the tolerant genotype of chickpea (*Cicer arietinum*) [69].

Influenced by gene expression and factors like DNA methylation, cold stress signals translate into physiological changes. Indeed, in chickpea and buckwheat (*Fagopyrum esculentum* Moench) [69,70], genes related to specific pathways were reported to correlate methylation status with expression. In particular, these genes are mainly involved in cellular metabolism, stress response, the lysine pathway, and the antioxidant system, but transcriptional regulation also showed the same correlation. Consistent with these observations, the *CMT2* gene exhibited downregulation, while genes such as *HbICE1* (*Inducer of CBF Expression 1*), *HbCBF2 (C-Repeat-Binding Factors 2),* and *HbMET*, known to be involved in enhancing DNA demethylation, showed strong upregulation instead [71].

However, cold adaptation requires changes in both methylation and demethylation. For instance, in Brassica, 1562 differentially methylated genes were identified during cold acclimation, including *BraDH1* (*Mitochondrial Malate Dehydrogenase1*), *BraKAT2* (*3-Keto-Acyl-CoA Thiolase-2*), *BraSHM4* (*Serine Hydroxy-Methyltransferase 4*), and *Bra4CL2* (*4-Coumarate-CoA Ligase 2*), which had demethylated promoters, resulting in increased transcriptional activity [72]. In the cold-tolerant rice variety P427, 51 genes characterized by methylation and expression changes were also identified. Interestingly, most of them were associated with the ICE–CBF–COR (Inducer of CBF Expression—C-repeat Binding Factor—Cold Regulation) pathway, already known to be important in the acquisition of cold tolerance [73]. Cold stress decreased DNA methylation in the promoter of the rice homologous gene of *OST1* (*Open Stomatal 1*) (Os03g0610900), which can interact with and phosphorylate *ICE1*, increasing its gene expression [73].

**Figure 1:**
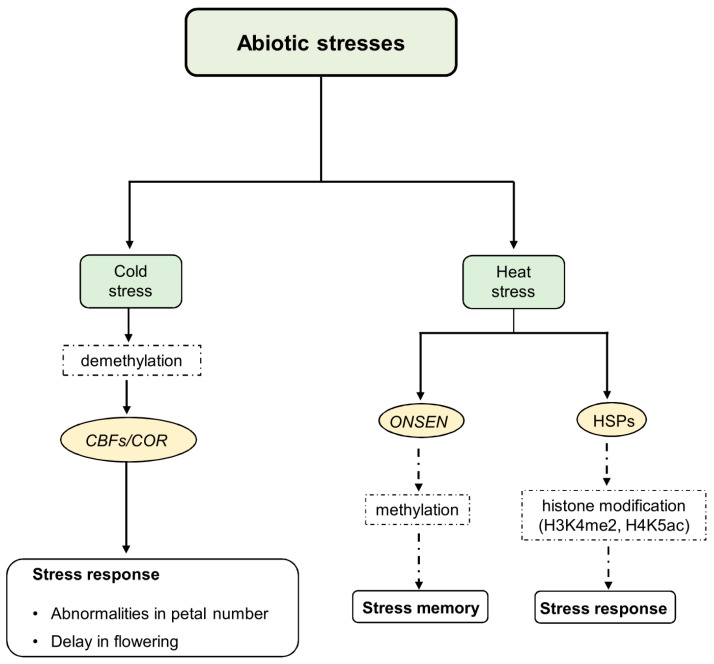
Epigenetic regulation of cold and heat stress response and memory abiotic stress signals induce changes in epigenetic regulators’ expression and/or function, including methyltransferases, chromatin remodeling factors, and enzymes responsible for histone modification. The cold response induces a decrease in both 5mC and 6mA levels to confer freezing tolerance. It involves the activity of CBF TFs that bind to COR promoters to activate COR gene expression. Under heat stress conditions, transcription of the ONSEN retrotransposon is induced, and HSPs are activated following histone modifications of HSP genes. Some of these changes result in permanent epigenetic modifications that can be inherited, while others result in transient changes. The transient changes in chromatin facilitate the acclimation response, while the permanent epigenetic changes contribute to stress memory within and between generations.

Several studies have detected significant changes in DNA methylation patterns in response to cold acclimation in plant species such as *Chorispora bungeana* [74], *Populus simonii* [75], and *Phyllostachys edulis* [76]. Cold acclimation is associated with DNA methylation and demethylation, with demethylation being particularly important for conferring freezing tolerance in *Arabidopsis*. In this context, reduced activity of the key RNA-directed DNA methylation (RdDM) pathway, which is important for both CHH and TEs methylation and its components, such as MSH1 (MutS Homologue 1) and RDM4, hinders the cold response in *Arabidopsis*. Furthermore, RDM4 controls the expression of CBF transcription factors, which are essential for developing and maintaining cold transcriptional memory [77,78] (Figure 1). Indeed, 5mC plays a role in modulating the ICE1-CBF-COR pathway, which is crucial for cold tolerance. For instance, in *Hevea brasiliensis*, the expression of *HbICE1* and *HbCBF2* is associated with DNA demethylation and enhanced cold tolerance [71]. In *Rosa hybrida*, cold-induced DNA methylation in the CHH context downregulates the expression of the *RhAGAMOUS* gene (*RhAG*), an *AGAMOUS (AG)* homolog that influences petal number induction [79]. Indeed, cold-based treatment in this species led to abnormal flowers with more petals than the wild type (Figure 1).

Moreover, it has been observed that both 5mC and 6mA levels decrease in response to cold stress [80]. However, the interaction between these two epigenetic marks in the context of freezing tolerance is not yet well understood. Low temperatures were associated with the hypermethylation of gene bodies, potentially leading to the repression of their expression [68]. DNA demethylation also plays a role in cold responses. Cold treatment increased the transcriptional activity of both cold-related and cold-responsive genes. Certainly, in rubber tree (*Hevea brasiliensis*) [71], *Brassica rapa* [72,81], tomato [82], and sweet orange (*Citrus sinensis* L.) [83], the transcriptional activity of cold-related and cold-sensitive genes, such as *HbICE1* and *HbCBF2*, is increased by cold treatment. These findings are accompanied by a high expression of DNA-methylation-related genes and also induce DNA demethylation of their promoters. However, methylation levels in the context of CHH showed a reduction under cold stress, consistent with transcriptional downregulation of *CMT2* and upregulation of genes related to DNA demethylation [67]. In poplar (*Populus* L.) apex tissue, low genomic DNA methylation was found to correlate with a higher expression of the chilling-dependent *DEMETER-LIKE DNA demethylase 10* (*DML10*), regulating bud break [84].

## 3. DNA Methylation in Regulating Plant Response to Heat Stress

Heat stress significantly threatens plant cells by causing cellular damage, increasing ROS production, and inducing oxidative stress. These effects can lead to various plant morphology, physiology, and biochemistry changes, including leaf shedding, flower and fruit loss, and even plant death [85]. The plant response to heat stress is complex and varies among species and temperature ranges, typically between 28 and 48 °C. It involves hormonal signaling, transcriptional processes, and epigenetic modifications. Plants respond to heat stress by employing regulated cell death (RCD) mechanisms, such as apoptosis, necrosis, or ferroptosis pathways. These selectively eliminate specific cells in certain tissues to maintain homeostasis under heat stress conditions [86].

DNA methylation is crucial in regulating gene transcription during heat stress, particularly impacting basal heat tolerance and transgenerational memory in *Arabidopsis*. However, the precise role of DNA methylation in heat stress memory remains incompletely understood. Studies on epigenetic *Arabidopsis* mutants have highlighted the significant involvement of the RdDM pathway and HISTONE DEACETYLASE 6 (HDA6) in the transcriptional response to heat [87,88]. In *Brassica napus*, differential DNA methylation patterns have been observed between heat-tolerant and heat-sensitive genotypes, suggesting dynamic changes in response to heat stress [89]. Heat-induced demethylation in maize has been linked to the spliceosome pathway, indicating a regulatory role of DNA demethylation in heat responses through RNA splicing [90]. Moreover, heat stress can induce genome-wide DNA hypomethylation, affecting metabolic pathways related to sugars and ROS [91].

In *Arabidopsis*, heat-induced changes in DNA methylation have been noted in specific genes with transposon insertions in their promoters, regulated by NUCLEAR RNA POLYMERASE D1A (NRPD1A) and NRPD1B under heat stress conditions. The dynamic nature of DNA methylation during prolonged heat stress is evident as key DNA methyltransferases like METHYLASE1 (MET1) and CMT3 are downregulated [66]. The regulation of retrotransposons by DNA methylation is critical for heat stress memory in plants. For instance, in *Arabidopsis*, the retrotransposon ONSEN can be activated by CMT3 under heat stress, contributing to transgenerational stress memory [92,93]. In detail, ONSEN transcription is modulated under heat stress by CMT3, which prevents CMT2-mediated CHH methylation and H3K9me2 (Histone H3 Lysine 9 dimethylation) accumulation at ONSEN chromatin. These studies highlighted the role of CMT3 in heat-induced ONSEN activation and shed new light on the mechanism by which certain transposons survive in the host genome under stress [92,93].

Interestingly, a loss-of-function mutation in CMT3 resulted in increased CHH methylation at ONSEN. CHH methylation is controlled by CMT2, as shown by the significant reduction in CHH methylation in *cmt2* and *cmt2 cmt3* mutants, combined with elevated ONSEN transcription. In addition, the authors observed increased CMT2 binding to ONSEN chromatin in *cmt3* mutants compared to the wild type. This was associated with ectopic accumulation of H3K9me2 under heat stress. This evidence suggests a cooperative role for H3K9me2 and CHH methylation in preventing heat-induced ONSEN activation. In conclusion, this study provides new insights into how DNA methyltransferases regulate transcription under stress conditions by identifying a noncanonical role of CMT3 in transposon silencing [92,93]. In *Arabidopsis*, transposons are regulated by the RdDM machinery, suppressing chromatin modifications and preventing retrotransposon transgenerational memory [17,18]. A study was conducted on *Arabidopsis* to investigate the methylation levels of *AtSN1*, a *SINE-like retrotransposon*, in response to high temperatures. Thermal stress increased the methylation state of *AtSN1*, leading to decreased expression in the tested plants. This increase in *AtSN1* methylation levels was closely associated with *DRM2*, *NRPD1*, and *NRPE1* (*Nuclear RNA Polymerase V*) transcriptional levels. It is possible that the transcription levels of *AtSN1* can be influenced by the interaction between the RdDM pathway and other factors, such as transcriptional factors and/or chromatin remodelers. These findings highlight the importance of the RdDM pathway in regulating methylation levels in plants subjected to heat stress [92,93].

Several genes and transcription factors are known to be pivotal for heat tolerance across different plant species. In tomatoes, transcriptome sequencing has revealed thousands of genes involved in protein kinase, oxidoreductase, and hydrolase activities, which are up- or downregulated by heat stress [94]. Heat shock proteins (HSPs), acting as molecular chaperones, play a crucial role in protecting cells from heat stress and are regulated by heat shock transcription factors (HSFs) [95,96]. Specific genes like *HSP17.6C*, *HSP21*, and *HSP22*, categorized as small HSPs (sHSPs), are closely associated with histone modifications such as H3K4me3 (H3 Lysine 4 Trimethylation) and H3K27me3 (Figure 1). Histone demethylases of the Jumonji C domain-containing protein family (JMJ) are essential for balancing these modifications and maintaining heat stress memory [96,97]. In cotton (*Gossypium hirsutum*), high temperatures induce significant changes in histone modifications like histone H3 Lysine 4 Dimethylation (H3K4me2) and histone H4 Lysine 5 Acetylated (H4K5ac), correlating with transcriptome variations and the expression levels of heat-responsive genes [98] (Figure 1).

Heat stress also induces post-transcriptional gene silencing (PTGS), facilitated by SUPPRESSOR OF GENE SILENCING 3 (SGS3), regulated by Heat Shock Factor A2 (HSFA2), and RELATIVE OF EARLY FLOWERING 6 (REF6), thereby contributing to transgenerational stress memory [99,100]. Small RNA-regulated ARGONAUTE1 (AGO1) is crucial for heat-stress-induced transgenerational inheritance in *Brassica rapa* [101]. The bHLH TF (basic helix–loop–helix transcription factor) PIF4 (Phytochrome-Interacting Factor 4) plays a significant role in thermomorphogenesis under elevated temperatures. Chromatin remodelers and histone modifications regulate it. The RNA-binding protein FLOWERING CONTROL LOCUS A (FCA) is recruited to the YUCCA 8 (YUC8) promoter through interaction with PIF4, resulting in the dissociation of PIF4 from the YUC8 promoter, thereby affecting stress memory [102].

These interactions demonstrate the intricate role of HSPs, HSFs, JMJ genes, and various TFs in regulating heat stress memory. However, the precise mechanisms and crosstalk between these components require further investigation.

## 4. DNA Methylation in Regulating Plant Response to Drought Stress

In recent decades, drought, exacerbated by climate change, has emerged as the most significant environmental stressor affecting plant yield and growth rates [103,104]. Plants have developed complex mechanisms to respond to this challenge, with DNA methylation regulation playing a crucial role in gene expression. When exposed to drought stress, plants exhibit higher overall DNA methylation levels than their well-hydrated counterparts. For example, in the case of mulberry (*Morus alba*), the increase is 8.64%, whereas in poplar (*Populus trichocarpa*), it rises by 2.29% [105,106].

Extensive correlation analyses have revealed that DNA methylation has a multifaceted impact on gene expression under drought conditions. It is evident that DNA methylation exerts its influence through multiple regulatory pathways, whether directly or indirectly. Notably, drought-tolerant plant species exhibit a more stable methylome in response to water scarcity. Differentially methylated region (DMR)-related genes are primarily associated with stress responses, programmed cell death (PCD), and other pivotal pathways across various species, including rice (*Oryza sativa* L.), mulberry (*Morus alba* L.), mungbean (*Vigna radiata* L.), and maize (*Zea mays* L.) [57,106,107,108,109,110]. For example, in rice, a comprehensive in silico analysis revealed unique *Cytochrome P450* (*CYP450*) genes with varying methylation levels under drought stress [111]. Similarly, water-scarcity-associated cytosine methylation patterns were found in drought-sensitive and drought-tolerant apple (*Malus × domestica*) varieties [112].

**Figure 2:**
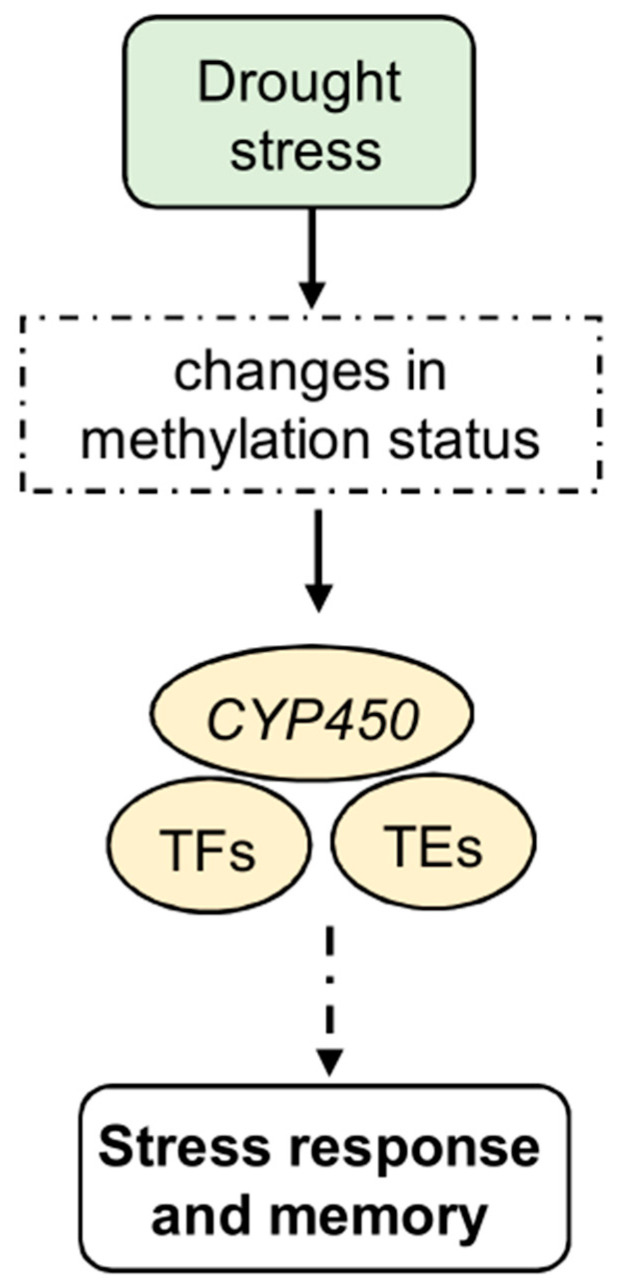
Epigenetic regulation of stress response, memory, and tolerance to drought stress. In particular, it induces changes in the methylation status of several genes, including *CYP450* genes. In addition, TFs, including some NACs, show a global decrease in methylation levels. Other TFs, such as WRKY and MYB, show altered expression levels after drought stress under TE control. Methylated transposons are often involved in the signaling pathways of several gene families known to be involved under abiotic stress conditions. These changes, which are involved in the stress response, may lead to the development of stress memory.

Global analyses of methylation and transcription patterns consistently demonstrate that genes with unmethylated promoters are expressed at higher levels than methylated ones. For example, in maize, DNA methylation within the *ZmNAC111(NAC-transcription factor 111)* promoter region lowers its levels of expression, rendering the plant more susceptible to drought [113]. In turn, gene body methylation changes in response to drought, affecting many genes, including those encoding TFs (Figure 2). Indeed, under water stress conditions, maize roots show a negative correlation between gene body methylation and gene expression [110]. Additionally, drought-induced changes in DNA methylation are not confined to genes but also extend to TEs (Figure 2). Methylated transposons implicated in drought signal transduction pathways have been identified in various gene families, such as *Zinc finger gene C2C2 type* (*C2C2*), *WRKY*, and *Myeloblastosis* (*MYB*), in *Populus trichocarpa* [106].

Crucially, two key types of enzymes, CYTOSINE-5-METHYLTRANSFERASES (C5-MTases) and demethylases, dynamically maintain the DNA methylation status of plant genomes under drought stress conditions. Drought stress prompts the upregulation of these enzymes, as observed in apple (*Malus × domestica*), tomato (*Solanum lycopersicum*), eggplant (*Solanum melongena* L.), and maize (*Zea mays* L.) [112,113,114,115,116,117].

Plants exhibit tissue-specific and genotype-specific DNA methylation characteristics under water-deficient conditions, resulting in variation and polymorphism.

Notably, drought-tolerant genotypes, like AK58 in wheat (*Triticum aestivum* L.), display higher levels of DNA methylation, demethylation, and increased methylation polymorphism than in common genotypes, such as MinMai 13 [118]. Furthermore, methylation polymorphisms are more pronounced in the roots than in the leaves under water-deficient conditions, especially in the drought-tolerant AK58 genotype. This result indicates that differential DNA methylation in the roots may contribute to the faster response to water deprivation demonstrated by AK58 [118].

DNA methylation plays a crucial role in response to drought stress by aiding stress memory and transgenerational epigenetic inheritance. Plants can ‘remember’ previous drought events through stable DNA methylation patterns, which helps them respond more efficiently to recurring stress conditions. Moreover, some of these DNA methylation changes can be inherited by the plant progeny, giving them an advantage in coping with water scarcity [119] (Figure 2).

As also reviewed in [120], manipulating DNA methylation can enhance stress tolerance in crops, offering exciting opportunities for crop improvement. Researchers are exploring this approach, potentially leading to the development of drought-resistant varieties.

Although our understanding of DNA methylation in drought response has advanced significantly, many questions remain unanswered. High-resolution epigenomic techniques and innovative research approaches are necessary to understand DNA methylation changes under drought stress [121].

## 5. DNA Methylation in Regulating Plant Response to Salt Stress

Soil salinization is a serious environmental problem threatening sustainable agriculture and food security [122]. High salinity levels dramatically affect critical plant processes, including osmotic and ionic balance, protein synthesis, photosynthesis, energy production, and lipid metabolism [123]. The growing body of evidence highlights the crucial role of DNA methylation in regulating gene expression responses to salinity stress [124].

Salinity stress can cause changes in DNA methylation status, which can vary depending on the plant species or specific genes affected by salt stress conditions [125]. For instance, in alfalfa (*Medicago* spp.), exposure to salinity leads to an increase in the plant methylome content. Still, alfalfa salt tolerance significantly decreases when seedlings are treated with 5-Azacitidine (5-AzaC, a DNA methylation inhibitor) [126]. Conversely, CG methylation levels in the epidermis genomic regions of *Arabidopsis* plants decrease significantly under NaCl-induced stress, with more pronounced reductions in severely stressed plants [127]. Salt-sensitive wheat genotypes show an increase in 5mC content, detected in CHG and CHH contexts in the shoot, whereas salinity-tolerant wheat cultivars like SR3 exhibit reduced 5mC levels [128]. In rice, tolerant genotypes display hypermethylation, while sensitive genotypes exhibit demethylation [129]. Moreover, distinct DNA methylation patterns are observed in rice and wheat root and shoot systems during salt stress [130,131]. Under salt stress, olive plants (*Olea europaea* subsp. *europaea* var. *europaea*) exhibit increased DNA methylation levels. Salt-tolerant cultivars, such as royal varieties, show more pronounced changes than Koroneiki cultivars [132]. These findings highlight the potential regulatory roles of DNA methylation in conferring salt tolerance, which may vary depending on the species and tissues.

In addition, in these conditions, DNA methylation actively regulates the expression of various genes, including those involved in membrane transport, heavy metal transport, and organic acid secretion. These genes, in turn, modulate stress signaling and elicit stress responses in plants. For example, NaCl-treated wheat plants showed a genotype- and tissue-specific increase in cytosine methylation in the high-affinity potassium transporters *TaHKT2;1 and TaHKT2;3,* resulting in enhanced salt tolerance [133]. Methylation of salinity-responsive genes, like flavonol synthase genes *TaFLS1* and *TaWRS15* in wheat (*Triticum aestivum*) and barley (*Hordeum vulgare*), can induce salinity tolerance in plants [134].

DNA demethylation also plays a pivotal role in salt stress responses. It was observed in *Nicotiana tabacum* that demethylation mediated by the overexpression of *AtROS1* (*Repressor of Silencing 1*) from *Arabidopsis* under salt stress conditions induces the expression of genes involved in flavonoid and antioxidant biosynthesis pathways [135] (Figure 2). Under salinity stress, the salt-tolerant rice variety Nonabokra shows high gene expression that encodes *Abscisic Acid-Responsive Element Binding Factor 8* (*OsBZ8*). In contrast, the sensitive variety IR64 displays a loss of DNA methylation at the *OsBZ8* locus [136]. Notably, most gene promoters displaying methylation changes in *Arabidopsis* progeny are hypermethylated under salt stress, resulting in lower gene expression levels [137]. Furthermore, the presence of cytosine modifications in both the untranslated regions and exons of rice under salinity stress highlights the important function of gene methylation in regulating gene expression [138].

**Figure 3:**
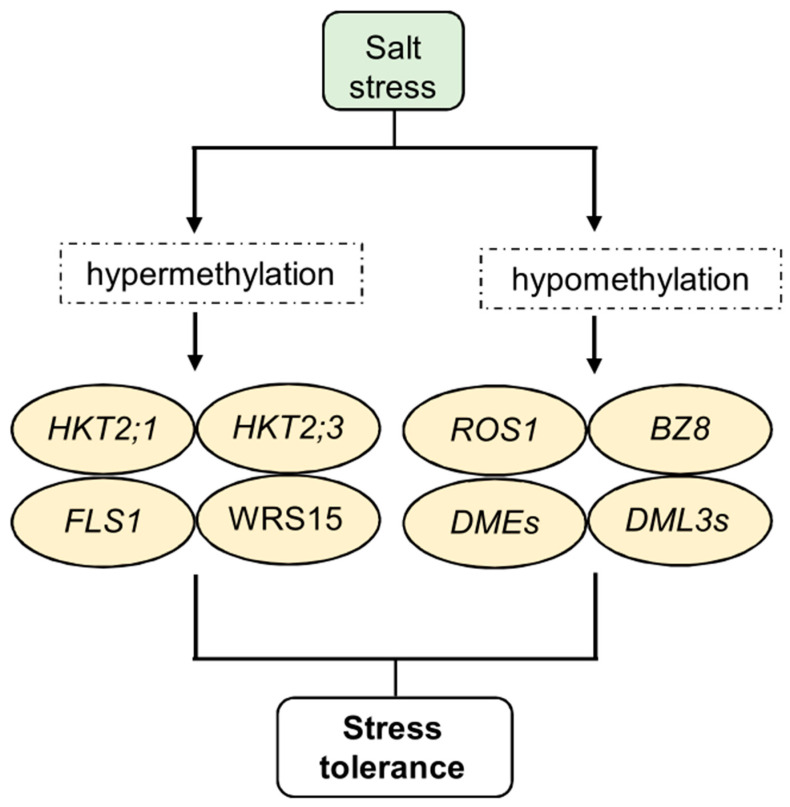
Epigenetic regulation of salt stress response, memory, and tolerance. In particular, plants exposed to salt stress show hypermethylation and hypomethylation of specific target genes. Cytosine hypermethylation was observed in the potassium transporters HKT2;1 and HKT2;3 and in the flavonol synthase genes *FLS1* and *WRS15*. In addition, demethylase genes such as *ROS*, *DML*, *DME*, and *BZ8* undergo strong changes in their expression. Some of these changes confer increased stress tolerance, which future progeny can inherit.

Salt stress significantly affects the expression of important enzymes involved in DNA methylation processes, such as CMT, DNMTs, DRMs, DEMETER (DMEs), and DMLs, leading to changes in plant DNA methylation. For example, some members of the CMT and MET families are significantly downregulated in response to salt stress, while *DNMT2* is upregulated in rapeseed (*Brassica napus* L.) [139]. Under salt stress, genes in the *DRM* family are generally downregulated. However, in *Brassica napus,* specific *DRM* genes, such as *BnaDRMa*, *BnaDRMg*, and *BnaDRMh*, show significant upregulation following salt treatment. Demethylase genes, including *DMEs*, *DML3s*, and *ROS1*, are mildly upregulated in rapeseed [139] (Figure 3). Additionally, the expression of *DME* responds to salt stress in a tissue-specific manner in *Pyrus betulaefolia*, with downregulation in leaves and upregulation in roots [140].

It is important to note that the impact of different single salt treatments and their combinations on plants may vary. Studies have revealed that the salt effects on DNA methylation in the halophyte *Chloris virgata* are as follows: Na_2_CO_3_ > NaHCO_3_ > Na_2_SO_4_ > NaCl, and mixed salts exhibit tissue-specific effects. This result underscores the complexity of plant responses to mixed salt treatments, not merely additive combinations of single salts [141].

Growing concern over soil salinization, which poses a serious threat to sustainable agriculture and global food security, has prompted intensive research into the potential applications of understanding DNA methylation in salt stress response. High salinity levels are known to have detrimental effects on fundamental plant processes, including disruption of osmotic and ionic balance, impairment of protein synthesis, inhibition of photosynthesis, impairment of energy production, and disruption of lipid metabolism [142]. These adversities ultimately translate into reduced crop yields and food shortages, particularly in regions struggling with salinity-induced soil degradation.

## 6. DNA Methylation Plays a Role in Regulating Plant Responses to Heavy Metal Stress

**Figure 4:**
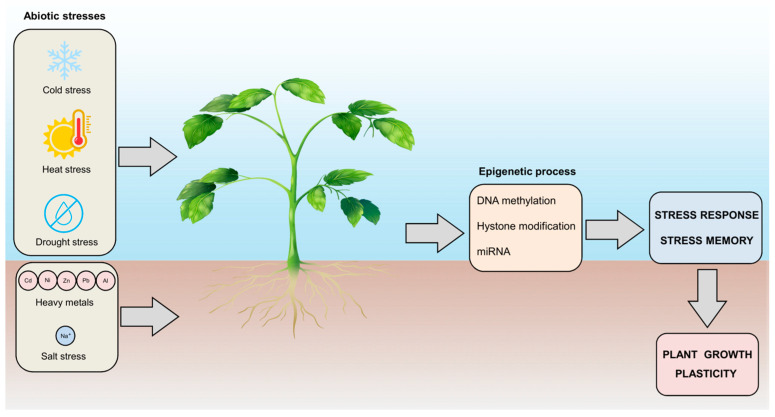
Plant responses to environmental stresses and the role of epigenetic control in plant growth plasticity. Plants are exposed to various environmental stresses that can affect growth and productivity. This exposure leads to plant stress responses and epigenetic regulation, which includes DNA methylation, histone modification, and miRNA regulation, which allows plants to attain stress memory. Understanding these fascinating features helps to identify targets for crop improvement.

The advent of industrial pollution has altered the equilibrium of certain heavy metals, causing their overabundance in soil, a detrimental circumstance, as revised in [143], for most plant species. Under such conditions, plant roots act as sponges, absorbing excess heavy metal ions from their surroundings and transporting them to aerial parts, disrupting their metabolic processes and hindering their growth [144]. Heavy metal stress is intrinsically associated with physiological responses, gene expression patterns, and DNA methylation profiles [145]. Consequently, there has been a concerted effort to unravel how plants protect themselves against the adverse effects of heavy metal stress (Figure 4).

Hypermethylation was identified as a defense mechanism plants employ to protect themselves from potential harm caused by heavy metal exposure, allowing them to thrive in the most challenging environments. On the other hand, it was also observed that the *Arabidopsis ddc* triple mutant has better growth performance than wild-type plants when exposed to cadmium (Cd) [146], highlighting the important role of methylation status under heavy metal exposition. In rice (*Oryza sativa* ssp *japonica* cv. Nipponbare), following exposure to Cd stress, a notable surplus of hypermethylated genes was observed compared to hypomethylated ones [147]. Also, in radish (*Raphanus sativus*) and soybean (*Glycine max*), after Cd exposure, an increase in methylation levels was observed, providing new evidence about the relationship between the two events and suggesting a dose-dependent response [148,149].

In addition, it was observed that in the roots of heavy-metal-tolerant plants, the methylation levels are higher than their sensitive counterparts. For instance, when comparing clover (*Trifolium repens* L., a sensitive species) with hemp (*Cannabis sativa* L., partially tolerant), hemp exhibits significantly higher methylation levels [150]. Similar distinctions are noted between Ni-tolerant *Noccaea caerulescens* and Ni-susceptible *Arabidopsis thaliana*, between which *Noccaea caerulescens* displays high methylation levels [151].

However, a different trend emerges in the case of the wheat-resistant variety, exposed to lead (Pb), Cd, and zinc (Zn), which exhibits DNA hypomethylation in the promoter regions of metal-detoxification transporters [152].

Notably, aluminum (Al) exposure induces diverse methylation responses. Indeed, in general, exposure to Al induces demethylation, except for *Zea mays*, which triggers DNA hypermethylation as a protective response, revealing the complexity of plant reactions to Al stress [153]. Plants of *Triticale*, an artificial hybrid of wheat (*Triticum aestivum*) and rye (*Secale cereale*), further illustrate this complexity because Al-tolerant lines show an increase in DNA methylation content. In contrast, nontolerant lines show a decrease [154]. These complex response patterns suggest that different mechanisms are at work, depending on factors such as the importance of the heavy metal element for plant growth, the plant species, and the stage of development.

However, regulation by DNA methylation under heavy metal stress is not restricted to the promoter regions of genes but also their coding regions and TEs. TEs have been implicated in Al stress responses in barley and were found to be highly methylated in radish under Pb treatment [155]. In addition, heavy metals induce both DNA methylation and demethylation processes. Indeed, an upregulation of *CMT1* associated with DNA hypermethylation was observed in *Posidonia oceanica* following Cd treatment [156]. Furthermore, mutations in *MET1* and *DRM2* were observed in rice seedlings grown under Cd stress, resulting in reduced transcript levels of *OsIRO2 (Fe-Deficiency-Inducible Basic Helix-Loop-Helix Transcription Factor2)* and *OsPR1b (Pathogenesis-Related Class 1 Genes B)*, highlighting the role of DNA methylation in the response to Cd [147]. Interestingly, recent evidence has shown that Zn, Pb, and Cd can differentially regulate the expression of DNA methyltransferases and DNA methylation levels in maize, individually or in combination [157].

## 7. Future Outlooks

The accumulating evidence on DNA methylation’s role in plant stress responses unveils many potential applications for crop improvement.

Insights into DNA methylation patterns under abiotic stresses can inform targeted breeding programs [158]. Breeders can develop crop varieties with specific DNA methylation patterns associated with improved abiotic stress tolerance, creating varieties that thrive in different environmental conditions. The emerging field of epigenetic editing holds great promise for modifying crop DNA methylation patterns [158].

Tools like CRISPR (clustered regularly interspaced short palindromic repeats)-based technologies can potentially modify DNA methylation marks in critical genes to improve abiotic stress tolerance, bypassing the need for lengthy traditional breeding programs [159,160,161,162].

For example, DNA methylation markers associated with salt tolerance can be employed for marker-assisted selection (MAS) in crop breeding. This approach speeds up the identification of salt-tolerant individuals, streamlining the breeding process and accelerating the development of resilient crop varieties [163,164,165].

Understanding the epigenetic regulation of stress-responsive genes allows researchers to identify key regulatory elements. This knowledge can be used to develop genetic engineering strategies to enhance stress tolerance in crops, not only to salinity and drought but also to other environmental stressors [163,164,165].

Farmers can use information on DNA methylation responses to salt stress and drought to adapt their growing practices. This may include adjusting irrigation schedules, nutrient management, or employing salt-tolerant rootstocks in horticultural practices to mitigate the adverse effects of salinity and water scarcities.

Genetic modification techniques can introduce genes or regulatory elements that promote DNA demethylation or maintain beneficial DNA methylation patterns under salt stress. Transgenic crops with enhanced salt tolerance could help address food security challenges in salt-affected regions.

A deeper understanding of DNA methylation’s role in abiotic stress response can inform crop diversification strategies. Farmers can select crop species or varieties with innate abiotic tolerance based on their epigenetic profiles, expanding agricultural options in different environmental areas [163,164,165].

Beyond agriculture, for example, insights into DNA methylation responses to salinity stress can aid in restoring salt-affected ecosystems. Conservationists and land managers can use this knowledge to select and propagate native plant species better equipped to thrive in saline environments during habitat-restoration efforts [163,164,165].

Elucidating the role of DNA methylation in plant responses to abiotic stresses has immense potential to revolutionize agriculture and ecological restoration. By harnessing this knowledge, we can develop innovative strategies to mitigate the effects of soil salinization, enhance crop resilience, and ensure future food security in an increasingly challenging environmental landscape.

## 8. Conclusions

This review addresses the multifaceted role of DNA methylation in plant responses to various environmental stressors, such as cold, heat, drought, salinity, and heavy metals. DNA methylation, a pivotal epigenetic modification, facilitates the regulation of gene expression without altering the underlying DNA sequence, thus enabling plants to dynamically adapt to changing environmental conditions (Figure 4). This capacity for adaptation is essential for plant growth, development, and survival, particularly in increasingly erratic and extreme environmental conditions driven by climate change. Our detailed examination of the mechanisms by which DNA methylation influences plant stress responses reveals a complex interplay between methylation patterns and other epigenetic processes, including histone modifications and RNAi (Figure 4). These interactions are crucial for fine-tuning gene expression in response to stress. For instance, DNA methylation can activate or repress specific genes involved in stress responses, thereby modulating physiological and developmental processes that enhance plant resilience. Several case studies across diverse plant species have illustrated the critical role of DNA methylation in environmental adaptation. For example, in *Arabidopsis thaliana*, mutations affecting DNA methylation pathways result in altered stress responses, demonstrating the importance of precise epigenetic regulation. Similarly, crop species such as rice, maize, and wheat exhibit distinct methylation changes in response to drought, salinity, and heat stress, highlighting the relevance of these mechanisms in agricultural contexts. This review also underscores the potential for leveraging our understanding of DNA methylation to improve crop resilience. By manipulating methylation patterns through breeding programs, epigenetic editing, or biotechnological interventions, it is possible to develop crop varieties that are better equipped to withstand environmental stressors. This approach offers a promising strategy for enhancing agricultural sustainability and ensuring food security in the face of global environmental challenges. In summary, the insights gained from studying DNA methylation in plant stress responses provide a valuable foundation for developing innovative agricultural practices. By harnessing the power of epigenetics, we can create crops that are more resilient to stress, thereby supporting sustainable farming and securing food supplies for future generations.

Understanding and manipulating DNA methylation can significantly enhance plant resilience to environmental stressors. This knowledge is key to developing stress-tolerant crop varieties, promoting sustainable agriculture, and ensuring food security in an increasingly unpredictable climate.

## Data Availability

In this work, no new data were created.

## Abbreviations

5mC, 5-methylcytosine; 6mA, N6-methyldeoxyadenosine; APA, Alternative Polyadenylation; AS, Alternative Splicing; CRISPR, Clustered Regularly Interspaced Short Palindromic Repeats; CYP450, Cytochrome P450; DMRs, Differentially methylated regions; ESCs, Embryonic Stem Cells; HSFs, Heat Shock Transcription Factors; HSPs, Heat Shock Proteins; MAS, Marker-Assisted Selection; MBDCap, Methyl CpG-Binding Domain-Based Proteins; MEDIP, Methylated DNA Immunoprecipitation; miRNA, microRNA; MSAP, Methyl-Sensitive Amplification Polymorphism; PCD, Programmed Cell Death; PTGS, Post-Transcriptional Gene Silencing; RCD, Regulated Cell Death; RNAi, RNA interference; sHSPs, Small Heat Shock Proteins; TEs, Transposable Elements; TGS, Transcriptional Gene Silencing; WGBS, Whole Genome Bisulfite Sequencing.
